# *Zfp148* Deficiency Causes Lung Maturation Defects and Lethality in Newborn Mice That Are Rescued by Deletion of p53 or Antioxidant Treatment

**DOI:** 10.1371/journal.pone.0055720

**Published:** 2013-02-06

**Authors:** Volkan I. Sayin, Anna Nilton, Mohamed X. Ibrahim, Pia Ågren, Erik Larsson, Marleen M. Petit, Lillemor Mattsson Hultén, Marcus Ståhlman, Bengt R. Johansson, Martin O. Bergo, Per Lindahl

**Affiliations:** 1 Wallenberg Laboratory, Institute of Medicine, The Sahlgrenska Academy at University of Gothenburg, Gothenburg, Sweden; 2 Department of Biochemistry, Institute of Biomedicine, The Sahlgrenska Academy at University of Gothenburg, Gothenburg, Sweden; 3 Sahlgrenska Cancer Center, Institute of Medicine, The Sahlgrenska Academy at University of Gothenburg, Gothenburg, Sweden; Virginia Commonwealth University, United States of America

## Abstract

The transcription factor Zfp148 (Zbp-89, BFCOL, BERF1, htβ) interacts physically with the tumor suppressor p53 and is implicated in cell cycle control, but the physiological role of Zfp148 remains unknown. Here we show that *Zfp148* deficiency leads to respiratory distress and lethality in newborn mice. *Zfp148* deficiency prevented structural maturation of the prenatal lung without affecting type II cell differentiation or surfactant production. BrdU analyses revealed that *Zfp148* deficiency caused proliferation arrest of pulmonary cells at E18.5–19.5. Similarly, *Zfp148*-deficient fibroblasts exhibited proliferative arrest that was dependent on p53, raising the possibility that cell stress is part of the underlying mechanism. Indeed, *Zfp148* deficiency lowered the threshold for activation of p53 under oxidative conditions. Moreover, both *in vivo* and cellular phenotypes were rescued on *Trp53* ^+/−^ or *Trp53* ^−/−^ backgrounds and by antioxidant treatment. Thus, Zfp148 prevents respiratory distress and lethality in newborn mice by attenuating oxidative stress–dependent p53-activity during the saccular stage of lung development. Our results establish Zfp148 as a novel player in mammalian lung maturation and demonstrate that Zfp148 is critical for cell cycle progression *in vivo*.

## Introduction

Zinc finger protein 148 (Zfp148, Zbp-89, BFCOL, BERF1, htβ) is a krüppel type transcription factor that binds to GC-rich DNA sequences, thus activating or repressing transcription of target genes [1]–[8]. Zfp148 targets represent a range of biological processes, however; the best characterized targets are associated with the cell cycle [5], [6], [8]–[10]. Those and other studies suggest a role for Zfp148 in cell cycle control, but attempts to establish the *in *vivo function of the protein have met with limited success.

*Zfp148* has been targeted three times in mice with inconsistent results [11]–[13]. Takeuchi reported that haploinsufficiency for *Zfp148* blocked germ cell differentiation in chimeric mice, thus precluding germline transmission of the targeted allele [13]. Another study suggested that *Zfp148*-deficient mice die at embryonic day (E) 9.5 with neural tube defects and anaemia [12]. Specific targeting of *Zfp148* exon 4, finally, caused postnatal lethality in homozygous mice and aggravated intestinal inflammation after dextran sulphate treatment [11]. Those discrepancies could reflect different targeting strategies, but the fact that all three papers report distinct phenotypes without apparent similarities underscores that the physiological role of Zfp148 remains poorly understood.

Cell culture experiments on the other hand strongly suggest that Zfp148 is involved in cell cycle control [5], [6], [8], [10], [14], [15]. For example, overexpressing Zfp148 in cancer cells induces growth arrest or apoptosis by regulation of *Cdkn1a* (*p21*) and *Bak*, respectively, and silencing Zfp148 induces senescence by regulation of *p16^ink4a^* [5], [6], [8]–[10]. Moreover, Zfp148 interacts with the tumour suppressor p53 and overexpression of Zfp148 increases p53 levels in the nucleus [14]. In consistency with those reports, transgenic expression of *Zfp148* in intestinal epithelium induced apoptosis and reduced intestinal tumour formation in the APC^Min/+^ model of intestinal cancer [16]. The physiological relevance of this finding is, however, not clear since the protein was highly overexpressed.

In this study, we generate *Zfp148*-deficient mice and show that Zfp148 plays an important and unexpected role in lung development. Zfp148 maintains proliferation of pulmonary cells during the saccular stage of lung development by suppressing oxidative stress–dependent p53 activity, thus preventing respiratory distress and lethality in newborn mice. The results demonstrate for the first time that Zfp148 controls cell proliferation *in vivo*, in a p53-dependent manner.

## Materials and Methods

### Mice

All animal experiments were approved by the animal research ethics committee in Gothenburg, Sweden. *Zfp148^+/gt^* mice were generated from ES cell clone XB878 (BayGenomics) and maintained on a 129P2/OlaHsd and C57Bl/6 mixed genetic background. *Trp53^tm1Tyj^ (Trp53^+/^*) mice (The Jackson Laboratory) were maintained on a 129Sv and C57Bl/6 mixed genetic background. PCR primers used for genotyping are listed in Supporting Information Table S2.

### Southern Blot Analysis

Genomic DNA was digested with *EcoR*I, *Hind*III, or *Kpn*I and analyzed with a *Zfp148* intron 4-specific probe using standard protocols.

### PCR Analyses

Primers are listed in Supporting Information Table S2.

### Real-time Quantitative PCR

TaqMan and Sybr Green assays were performed as described [17] using TaqMan/Sybr Green universal PCR mastermix (Applied Biosystems) and the pre-designed TaqMan assays (Applied Biosystems) or RT-PCR primers listed in Supporting Information Table S2.

### Western Blotting

Urea-dissolved total protein extracts were analyzed using antibodies recognizing Zfp148 (HPA001656), p16^ink4a^ (M-156, Santa Cruz Biotechnology), phospho-p53^Ser18^ (9284, Cell signalling Technology), and ACTIN (A2066, SigmaAldrich Atlas) as described [18].

### X-Gal Staining

E9.5 embryos were dissected and immediately fixed at 4°C for 2 hours in 0.2% glutaraldehyde and 1.5% formaldehyde and whole-mount stained at 37°C overnight as in [19].

### Immunohistochemistry

The primary antibodies anti-CC10 (Upstate Cell Signaling Solutions; 1∶500) and anti-cleaved caspase 3 (9661, Cell Signaling; 1∶750) were used for immunofluorescence and visualized with anti-rabbit IgG horseradish peroxidase-linked antibody (GE Healthcare; 1∶200), TSA Cyanine 3 System (PerkinElmer) or goat anti-rabbit Alexa 546 (Molecular Probes; 1∶500) in a Leica TCS SP5 confocal microscope or a Leica Image1 microscope (Leica Microsystems AG). BrdU positive cells were detected with 5-Bromo-2'-deoxy-uridine Labeling and Detection Kit I (Roche). Image analyses were done with BioPix iQ software (version 2.1.8., BioPix).

### Transmission Electron Microscopy

Lungs were fixed in 2% paraformaldehyde and 2.5% glutaraldehyde. Tissue slices were post-fixed in reduced osmium tetroxide and uranyl acetate, dehydrated, and embedded in epoxy resin. Ultrathin sections (Leica UC6 ultramicrotome) were examined in a LEO 912AB electron microscope.

### Surfactant Lipid Analysis

Lipids from lung tissue were extracted as described [20]. Levels of cholesteryl esters, triglycerides and free cholesterol were determined by HPLC [21]. Analysis of phospholipids was performed by mass spectrometry [22].

### DNA Synthesis

5-bromo-2'-deoxyuridine (BrdU, Calbiochem/Merck) (20 µmol/l) was added to the culture medium for 4 h and determined with an ELISA kit (Calbiochem/Merck). For *in vivo* BrdU labelling, pregnant mothers were injected with 150 mg/kg BrdU (Roche), and lungs from E19.5 embryos were collected 2 hours after injection.

### Cell Culture

Primary MEFs were isolated from E13.5–14.5 embryos and cell proliferation assays were performed as described [18]. When indicated, the culture medium was supplemented with 100 µmol l^−1^ NAC (Sigma Aldrich) and changed daily.

### Apoptosis

Apoptosis of cultured cells was evaluated with the Annexin V-EGFP Apoptosis Kit (K104–100, Biovision). Apoptosis in P1 lungs was evaluated by immunohistochemical staining with an antibody recognizing cleaved caspase 3 or with the TUNEL kit ApopTag® Fluorescence In Situ Apoptosis Detection Kit (Millipore).

### Adenoviral Transduction of MEFs

Cells were incubated with 15 multiplicities of infection of empty control adenoviruses (Ad*Null)* or adenoviruses encoding Zfp148 (Ad*Zfp148*) or Zfp148FLAG (Ad*Zfp148*FLAG) (Vector Biolabs) for 36–48 h before analyses.

### Cell-cycle Analysis

*Zfp148^gt/gt^* MEFs at passage 4 were trypsinized, fixed in 70% ethanol, incubated with propidium iodide and RNase A for 30 min at 37°C and analyzed in a FACScan flow cytometer with CellQuest Pro software (version 4.0.2, Becton Dickinson).

### NAC Treatment

Pregnant females of *Zfp148^+/gt^* intercrosses were administered NAC in the drinking water at a concentration of 1 g/l.

### Statistics

Values are mean ± SEM. Statistics were performed with two-tailed Student’s *t*-test for comparisons between two groups; one-way ANOVA with Tukey’s post-hoc test for multiple groups; two-way ANOVA for multiple groups and genotypes; log-rank test for survival; Chi-square test for genotype frequencies. Differences between groups were considered significant when *P*<0.05.

## Results

### Generation of *Zfp148*-deficient Mice

**Figure 1:**
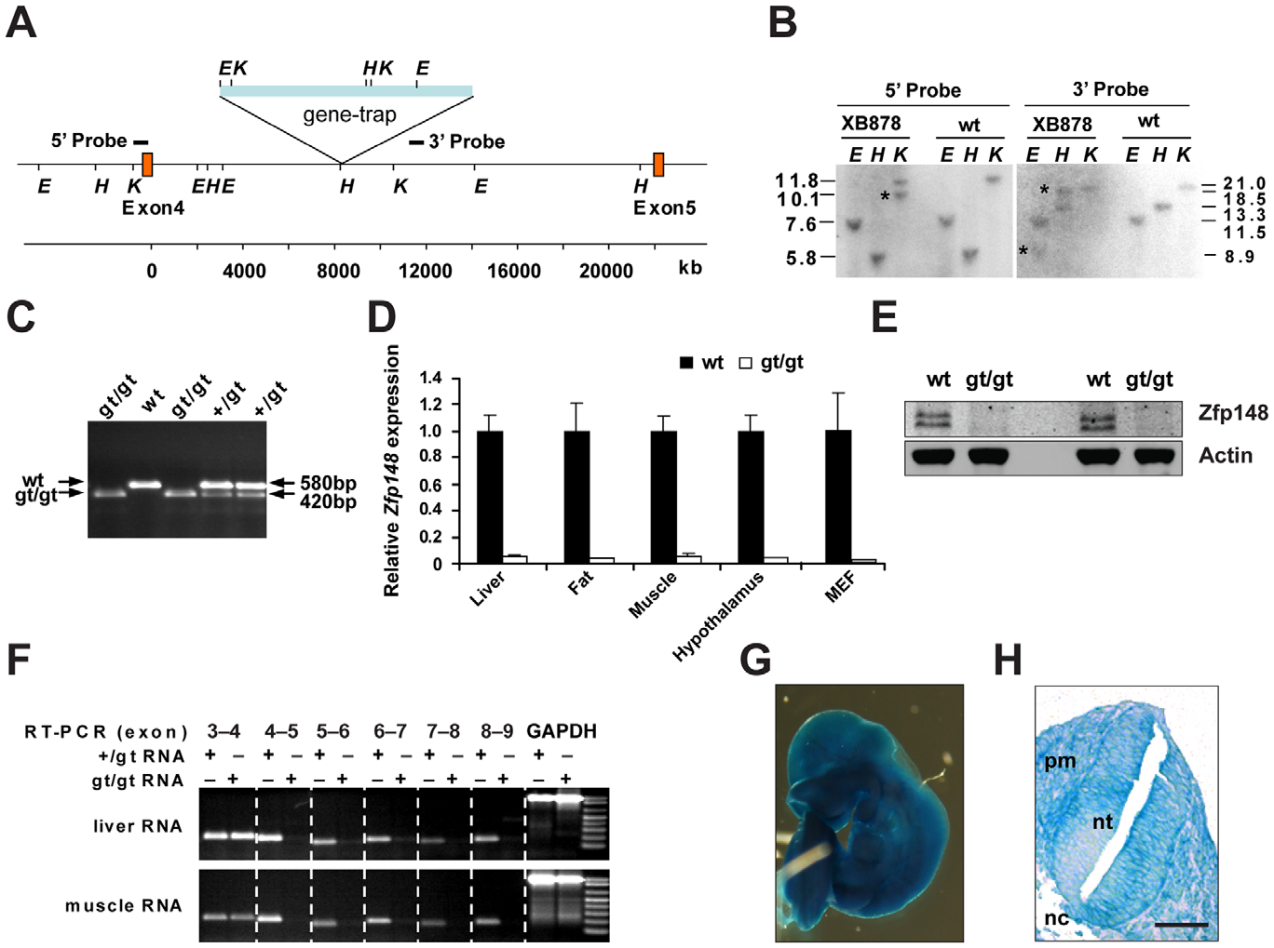
Generation of *Zfp148^gt/gt^* mice. (**A**) Map of the *Zfp148* locus indicating the gene-trap insertion site, restriction sites for *EcoRI* (*E*), *HindIII* (*H*) and *KpnI* (*K*), and target sites for Southern blot probes. (**B**) Southern blot analyses of DNA from XB878 ES-cells and wild-type controls, digested with the restriction enzymes *E*, *H*, and *K*, respectively (asterisk indicates mutant band). Fragment size in kb. (**C**) PCR amplification across the gene-trap insertion on tail DNA from pups of *Zfp148^+/gt^* intercrosses confirmed gene-trap insertion at approximately 8500 base-pairs (bp) downstream of exon 4. The gene-trap specific band of 420 bp was amplified using a forward primer at 8256 bp downstream of exon 4 and a reverse primer in the gene-trap. The wild-type band of 580 bp was amplified using the same forward primer and a reverse primer at 8813 bp downstream of exon 4. (**D**) Real-time RT-PCR of the exon 4–5 border on cDNA from tissues of adult *Zfp148^gt/gt^* and wild-type mice, respectively. (**E**) Western blots showing expression of Zfp148 in *Zfp148^gt/gt^* and wild-type mouse embryonic fibroblasts (MEFs). Actin was used as a loading control. (**F**) RT-PCR amplification across the indicated exon–exon borders of the *Zfp148* cDNA isolated from liver and muscle in *Zfp148^gt/gt^* and *Zfp148^+/gt^* mice did not indicate cryptic splicing of the mRNA or alternative transcription start sites. Amplification of GAPDH was included as a loading control. (**G**) Whole mount image of X-Gal stained *Zfp148^gt/gt^* mouse at E9.5. (**H**) Photomicrograph showing X-Gal stained transverse section of E9.5 *Zfp148^gt/gt^* mouse. Scale bars, 100 µm. Neural tube (nt), notochord (nc) and paraxial mesoderm (pm).

To define the *in vivo* and cellular importance of Zfp148, we generated *Zfp148*-deficient mice from a gene-trap ES-cell clone. Southern blot analyses and genomic PCR confirmed that the gene-targeting vector had incorporated into the fourth intron of *Zfp148*, thus disrupting 87% of the coding sequence including the DNA binding zinc finger domains (Fig. 1A–C). ES-cells were injected into C57Bl/6 blastocysts to achieve germline transmission of the *Zfp148^gt^*-allele. Heterozygous intercrosses of *Zfp148^+/gt^* mice revealed that *Zfp148* mRNA expression was reduced by >95% in *Zfp148^gt/gt^* tissues and that the protein was undetectable in western blot analyses (Fig. 1D, E). There were no indications of exon skipping or cryptic splicing (Fig. 1F). The inserted gene-trap vector harbours a promoterless β-Geo reporter, under control of the endogenous *Zfp148* promoter. X-Gal staining of embryonic day 9.5 (E9.5) *Zfp148^gt/gt^* mice suggested that *Zfp148* is ubiquitously expressed (Fig. 1G, H), in consistency with earlier results [2], [4].

### *Zfp148* Deficiency Leads to Lethality in Newborn Mice, and Growth Retardation and Reduced Lifespan in Adult Mice

**Figure 2:**
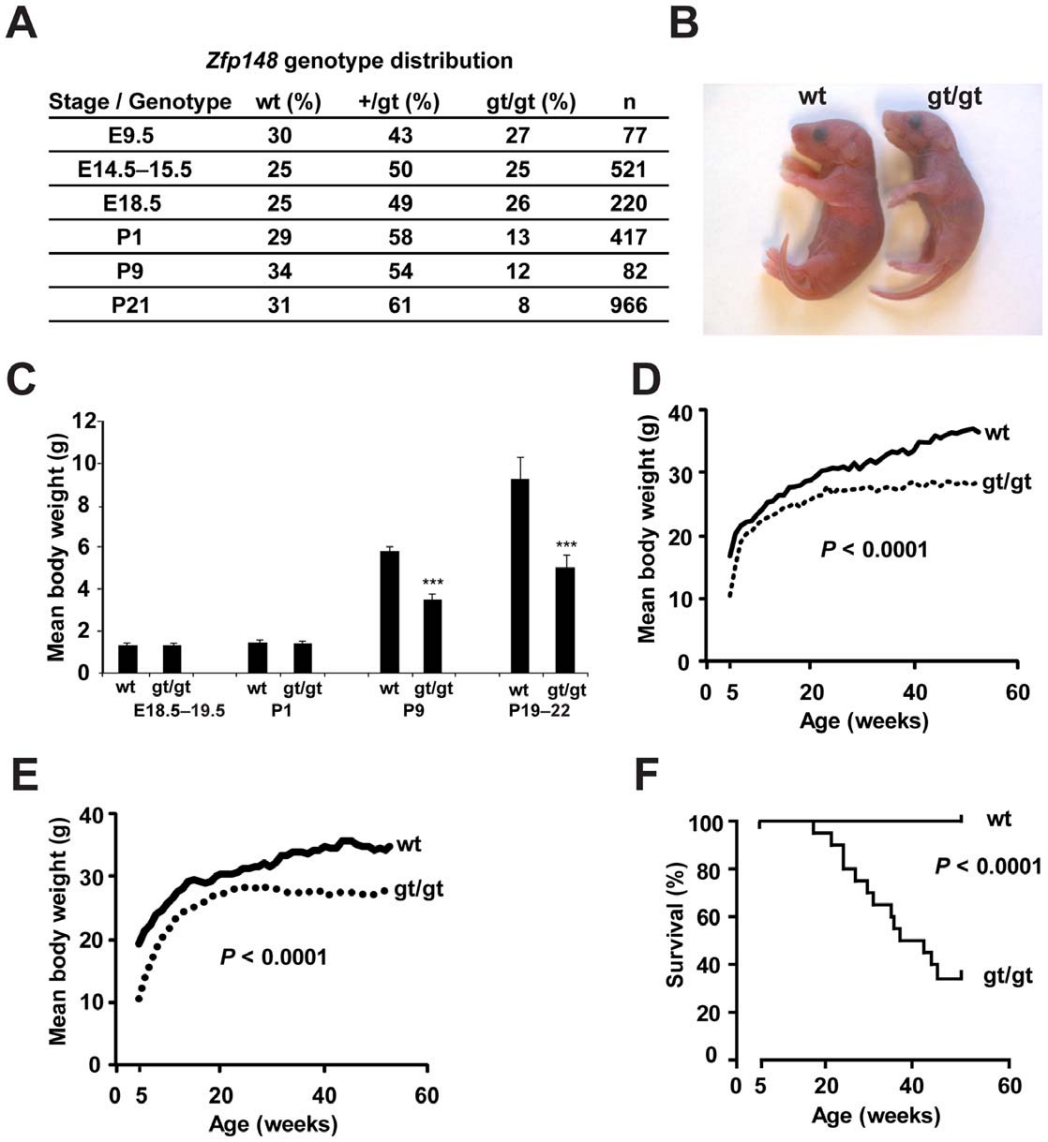
*Zfp148* deficiency causes lethality in newborn mice and growth retardation and reduced life span in adult mice. (**A**) *Zfp148*-genotype distribution of offspring from heterozygous intercrosses. (**B**) Photograph of cyanotic *Zfp148^gt/gt^* mouse and wt littermate at P1. (**C**) Body weight of wt, *Zfp148^+/gt^* and *Zfp148^gt/gt^* mice of mixed genders at E18.5–19.5 (*n* = 12 wt, 15 *Zfp148^+/gt^*, 8 *Zfp148^gt/gt^*), P1 (*n* = 9 wt, 13 *Zfp148^+/gt^*, 7 *Zfp148^gt/gt^*), P9 (*n* = 28 wt, 44 *Zfp148^+/gt^*, 10 *Zfp148^gt/gt^*) and P19–22 (*n* = 10 wt, 18 *Zfp148^+/gt^*, 9 *Zfp148^gt/gt^*). (**D, E**) Body weight curves for adult wt and *Zfp148^gt/gt^* male (D) and female (E) mice, respectively (*n* = 10). (**F**) Kaplan-Meier plots showing survival of *Zfp148^gt/gt^* and wt mice (*n* = 20). ***P<0.001.

*Zfp148^gt/gt^* mice were identified at Mendelian ratios throughout embryonic development, but the frequency dropped to half the expected at postnatal day 1 (P1) suggesting that 50% of the mice died shortly after birth (Fig. 2A). *Zfp148^gt/gt^* mice at postnatal day P1 were pale and cyanotic indicating respiratory problems (Fig. 2B). *Zfp148^gt/gt^* mice that survived the neonatal crisis were initially normal in size but displayed impaired growth and reduced lifespan (Fig. 2C–F). To specifically assess the postnatal importance of Zfp148, mice that were moribund at five weeks of age (study start) were excluded from the lifespan study. The study is therefore likely to underestimate mortality at young ages.

### Respiratory Distress in Newborn *Zfp148^gt/gt^* Mice is Caused by Defect Lung Maturation

**Figure 3:**
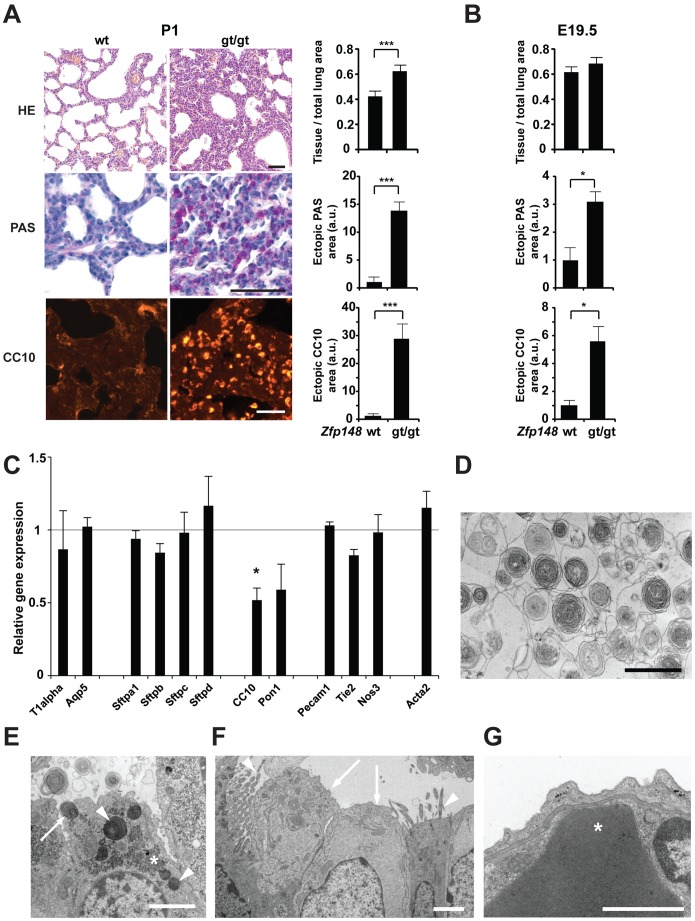
*Zfp148* deficiency prevented structural maturation of prenatal lungs without effecting epithelial cell differentiation or surfactant production. (**A**) Photomicrographs showing lung morphology (hematoxylin and eosin; HE) and glycogen content (periodic acid-Shiff; PAS) and CC10 immunofluorescence in P1 lungs from *Zfp148^gt/gt^* and wt mice, respectively. (**A, B**) Graphs show quantification (*n* = 6) of mean tissue area per total lung area, PAS-positive area with bronchioles excluded, and CC10-positive area with bronchioles excluded in (A) P1 and (B) E19.5 lungs from wt and *Zfp148^gt/gt^* mice. a.u., arbitrary units. (**C**) Real-time RT-PCR showing relative expression levels of markers for type I (T1alpha, Aqp5), type II (Sftpa1, Sftpb, Sftpc, Sftpd), clara (CC-10, Pon1), endothelial (Pecam1, Tie2, Nos3) and smooth muscle (Acta2) cells in *Zfp148^gt/gt^* lungs compared to wt at P1 (*n* = 6). Wt means are represented by the horizontal straight line at 1. (**D**) Transmission electron microscope (TEM) image of *Zfp148^gt/gt^* lung at E18.5–19.5 showing lamellar bodies secreted into the lumen of a terminal sac. (**E–G**) TEM images showing differentiated cells in *Zfp148^gt/gt^* lungs at E18.5–19.5. (E) Apical part of an alveolar type II cell containing typical lamellar bodies (arrowheads), one of which is in the process of exocytosis (arrow), and accumulations of densely contrasted glycogen particles (asterisk). (F) Two ciliated cells (arrowheads) surrounding two Clara cells (arrows) with bulging appearance and mitochondria accumulated in the apical cytoplasm. (G) High power view of the blood-alveolar barrier showing an erythrocyte (asterisk) in close contact with a highly attenuated part of an endothelial cell that shares a basal lamina with the alveolar type I cell. Scale bars, 2 µm. *P<0.05, ***P<0.001.

The cyanotic appearance of *Zfp148^gt/gt^* mice at P1 suggested problems with oxygenation. Histological analyses of lung tissue revealed poorly developed respiratory saccules with thickened primary septae, increased glycogen deposits and widespread ectopic expression of the Clara cell 10 kDa secretory protein CC10 (Fig. 3A). Analyses of lungs at E19.5 revealed that glycogen accumulation and ectopic CC10 expression occurred before birth (Fig. 3B). The results suggest a defect in sacculation, i.e. the transformation of distal lung buds into thin-walled terminal sacs that occurs at E18.5–P5 in mice.

Ectopic expression of CC10 protein indicated that cell differentiation could be disturbed in *Zfp148* ^gt/gt^ lungs. However, differentiation of type II cells appeared normal, as judged by the expression of surfactant-related genes, the presence of lamellar bodies, and levels of the surfactant lipid dipalmitoylphosphatidylcholine (Fig. 3C**–**E; Supporting Information Table S1). There was further no difference in the appearance of blood air barriers or expression of biomarker genes for type I cells (T1alpha, Aqp5) or endothelium (Pecam1, Tie2 Nos3) (Fig 3C, G). Notably, mRNA levels for the Clara cell markers CC10 and Pon1 were slightly reduced in *Zfp148^gt/gt^* lungs, in spite of the ectopic expression of CC10 protein, indicating that Clara cell differentiation could be affected, although the cells appeared morphologically normal (3C, F). We conclude that *Zfp148* deficiency prevents structural maturation of the prenatal lung without affecting type II cell differentiation or surfactant production.

### *Zfp148*-deficiency Disrupts Cell Proliferation at the Saccular Stage of Lung Development

**Figure 4:**
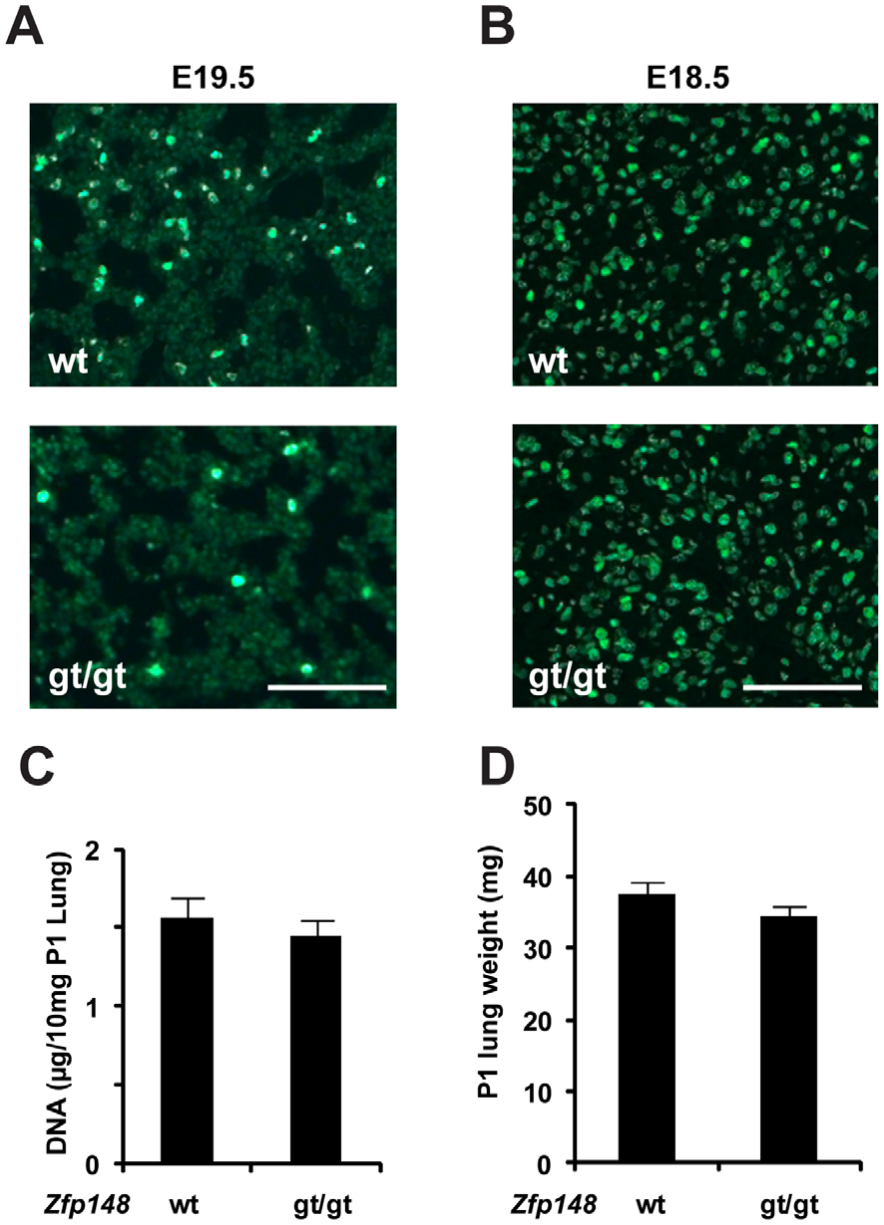
*Zfp148* deficiency reduced cell proliferation at the saccular stage of lung development. (**A, B**) Photomicrographs showing BrdU labeling of E19.5 (A) and E18.5 (B) lungs, respectively, from wt and *Zfp148^gt/gt^* mice. (**C**) DNA content per 10 mg lung tissue (*n* = 5). (**D**) Lung wet weight at P1 (*n* = 12). Scale bars, 100 µm.

Zfp148 is implicated in cell cycle control suggesting that *Zfp148^gt/gt^* lung defects could involve disturbed cell proliferation or apoptosis. To assess if cell proliferation was affected, E18.5 and E19.5 pregnant females were injected with 5-bromo-2′-deoxyuridine (BrdU). *Zfp148^gt/gt^* lungs at E19.5 contained fewer BrdU-positive cells compared to *Zfp148^+/gt^* lungs and controls, indicating problems with cell proliferation (Fig. 4A). There was no difference in BrdU incorporation at E18.5 between *Zfp148^gt/gt^* lungs and controls (Fig. 4B), suggesting that cell proliferation is interrupted during the saccular stage and that defective proliferation is coupled to the maturation defect. In consistency with those results, *Zfp148* deficiency did not affect total lung DNA content or lung wet weight at P1 (Fig. 4C, D), as would have been expected if proliferation was disturbed at earlier stages. There was no difference in apoptosis as judged by staining for activated caspase 3 or the TUNEL assay (Supporting Information Fig. S1).

### *Zfp148* Deficiency Arrests Cell Proliferation in a Cell Autonomous Manner

**Figure 5:**
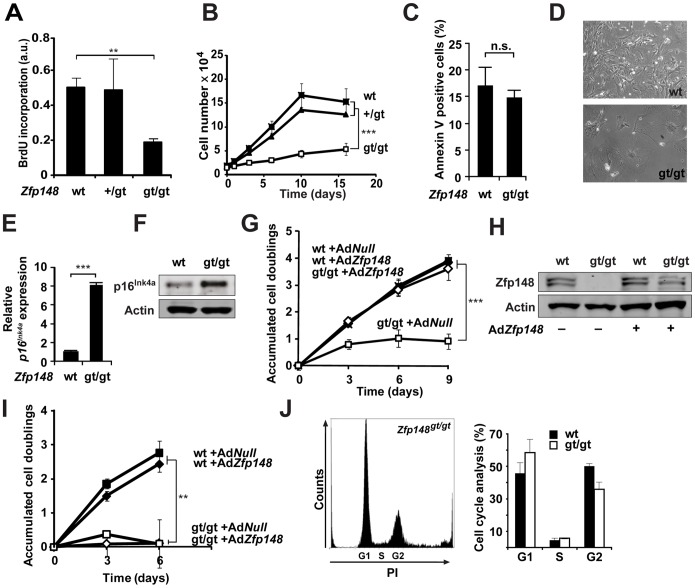
Proliferation arrest in *Zfp148^gt/gt^* MEFs. (**A**) Quantification of BrdU incorporation in wt, *Zfp148^+/gt^* and *Zfp148^gt/gt^* MEFs (*n* = 3). (**B**) Proliferation of *Zfp148^gt/gt^*, *Zfp148^+/gt^*, and wt MEFs (*n* = 4). (**C**) Apoptosis defined by FACS analyses of Annexin V in wt and *Zfp148* ^gt/gt^ MEFs at passage 2 (*n* = 3). (**D**) Photomicrographs of wt and *Zfp148^gt/gt^* MEFs at passage 2. (**E**) Real-time RT-PCR of *p16^ink4a^* mRNA in wt and *Zfp148^gt/gt^* MEFs (*n* = 4). (**F**) Western blot analysis of p16^ink4a^ protein in wt and *Zfp148^gt/gt^* MEFs. (**G**) Cumulative population doublings (CPD) of *Zfp148^gt/gt^* and wt MEFs transduced with *AdZfp148* or Ad*Null* at passage 0 (*n* = 3). (**H**) Western blot analysis of Zfp148 expression levels in wt and *Zfp148^gt/gt^* MEF cell lines transduced with Ad*Zfp148*. (**I**) CPD of *Zfp148^gt/gt^* and wt MEFs transduced with *AdZfp148* or Ad*Null* at passage 2 (*n* = 3). (**J**) Cell cycle profile of *Zfp148* ^gt/gt^ MEF (left) and quantification of cell cycle distribution of *Zfp148* ^gt/gt^ and wt MEFs (*n* = 5) (right) at passage 4 measured by FACS analysis of propidium iodide (PI)–labelled cells. **P<0.01, ***P<0.001.

To investigate mechanisms underlying the proliferation defect in *Zfp148^gt/gt^* lungs, mouse embryonic fibroblasts (MEFs) were isolated from E13.5–14.5 embryos of heterozygous intercrosses. *Zfp148^gt/gt^* MEFs proliferated poorly at early passages (Fig. 5A, B) but did not show increased apoptosis (Fig. 5C), compared to wildtype controls. *Zfp148^gt/gt^* MEFs exhibited morphological signs of senescence and expressed the senescence marker p16^ink4a^ (Fig. 5D–F), in consistency with a previous study [8]. The proliferation arrest of *Zfp148^gt/gt^* MEFs was abolished by restoring near physiological expression levels of Zfp148 by adenoviral transduction (*AdZfp148*) at passage 0 (Fig. 5G, H). *AdZfp148* transduction at passage 2 did not reverse the proliferation arrest (Fig. 5I), suggesting that cell cycle arrest is irreversible once established. *Zfp148^gt/gt^* MEFs were arrested regardless of their position in the cell cycle, as judged by cell cycle analysis at passage 4 (Fig. 5J).

### *Zfp148* Deficiency Lowers the Threshold for p53 Activation and Proliferative Arrest under Oxidative Conditions

**Figure 6:**
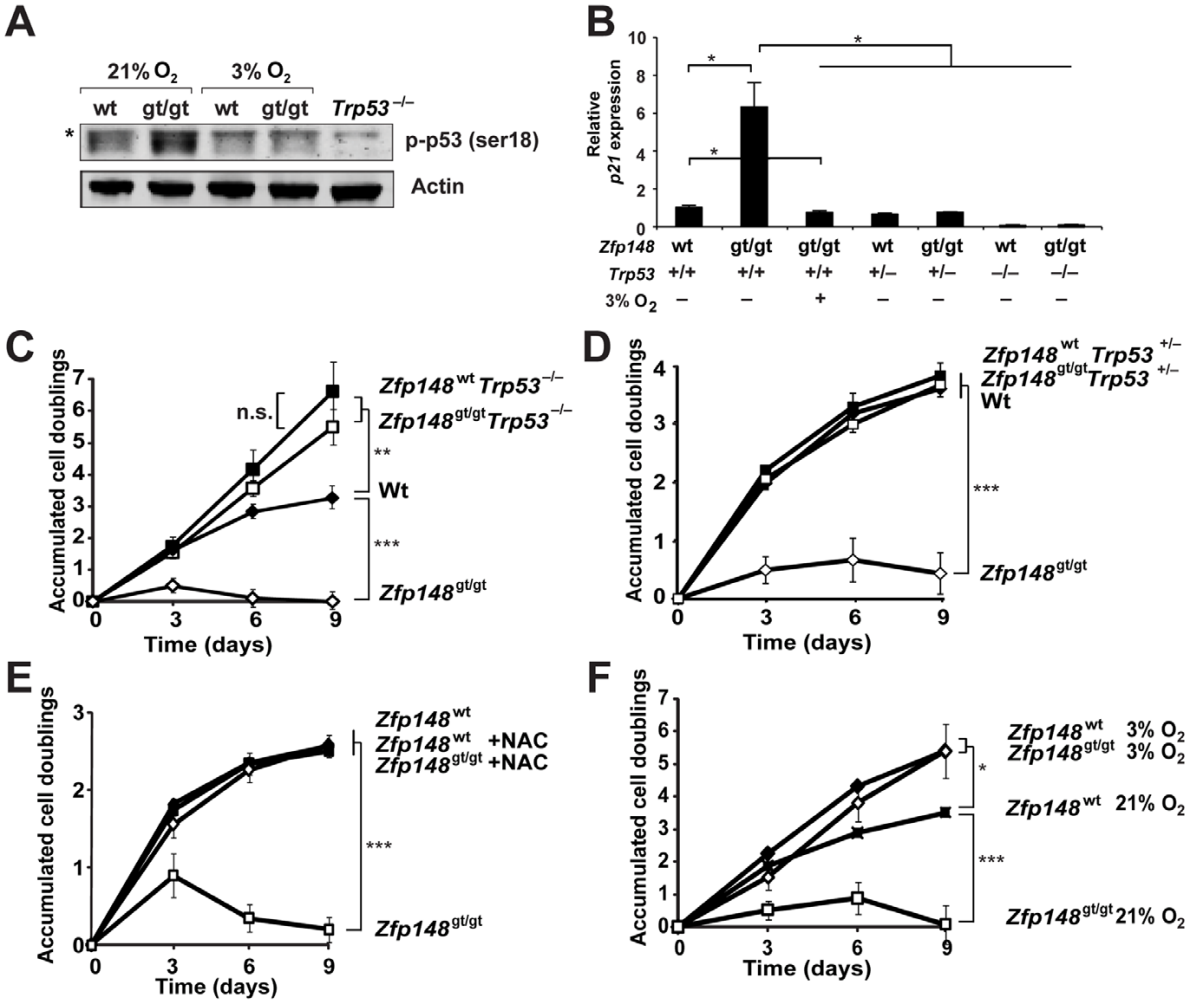
Activation of p53 and *Trp53*-dependent proliferation arrest in *Zfp148^gt/gt^* MEFs. (**A**) Western blots of MEF lysates showing expression of phospho-p53^Ser18^ in wt and *Zfp148^gt/gt^* MEFs cultured at 21 or 3% oxygen, respectively. *Trp53^−/−^* cells were used as a negative control and actin was used as a loading control. Asterisk indicates unspecific band. (**B**) Real-time RT-PCR of *p21* in wt and *Zfp148^gt/gt^* MEFs cultured at 21 or 3% oxygen, respectively, and on *Trp53^+/+^*, *Trp53^+/−^* and *Trp53^−/−^* genetic backgrounds (*n* = 4). (**C, D**) CPD of *Zfp148^gt/gt^* and wt MEFs on *Trp53^+/+^* or *Trp53^−/−^* (C) and *Trp53^+/+^* or *Trp53^+/−^* (D) genetic backgrounds (*n* = 3). (**E, F**) CPD of *Zfp148^gt/gt^* and wt MEFs supplemented with *n*-acetyl-L-cysteine (NAC) (E) in the culture medium (*n* = 4), or cultured at atmospheric (21%) or low (3%) oxygen concentrations (F) (*n* = 3). *P<0.05, **P<0.01, ***P<0.001.

*Zfp148* deficiency is not likely to arrest cell proliferation under physiological conditions, since *Zfp148^gt/gt^* embryos develop to term without gross malformations. The tumour suppressor p53 induces proliferative arrest in response to a wide range of stressors, including oxidative stress [23], [24]. The interaction of Zfp148 with p53 opens up for the possibility that *Zfp148* deficiency lowers the threshold for p53-activation. To assess this possibility, levels of phosphorylated p53 were measured under oxidative and non-oxidative conditions. We observed increased levels of phospho-p53^Ser18^ (p53^Ser 15^ in humans) protein in *Zfp148^gt/gt^* MEFs cultured at 21% oxygen, but not at 3% oxygen (Fig. 6A). Similarly, levels of mRNA for the p53-target *p21* were increased in *Zfp148^gt/gt^* MEFs cultured at 21% oxygen, but not at 3% oxygen (Fig. 6B).

To assess if the proliferation arrest of *Zfp148^gt/gt^* MEFs is mediated by p53, cells were obtained from *Zfp148^gt/gt^* mice and controls, bred on a *Trp53* null background. Loss of one or two copies of *Trp53* abolished proliferative arrest in *Zfp148^gt/gt^* MEFs (Fig. 6C, D and Supporting Information Fig. S2). In line with those results, induction of p21 was abolished in *Zfp148^gt/gt^* MEFs on *Trp53^+/−^* or *Trp53^−/−^* background (Fig. 6B).

The selective activation of p53 in *Zfp148*-deficient MEFs cultured at 21% oxygen, suggest that oxidative stress is part of the underlying mechanism. To determine if oxidative stress contributes to the proliferative arrest, *Zfp148^gt/gt^* MEFs were cultured in the presence of the antioxidant *n*-acetyl-L-cysteine (NAC) or at low oxygen concentrations (3%). Both treatments prevented the proliferative arrest (Fig. 6E, F).

### Deletion of One or Two Copies of *Trp53* Rescued *Zfp148^gt/gt^* Mice from Proliferation Arrest, Respiratory Distress and Neonatal Lethality

**Figure 7:**
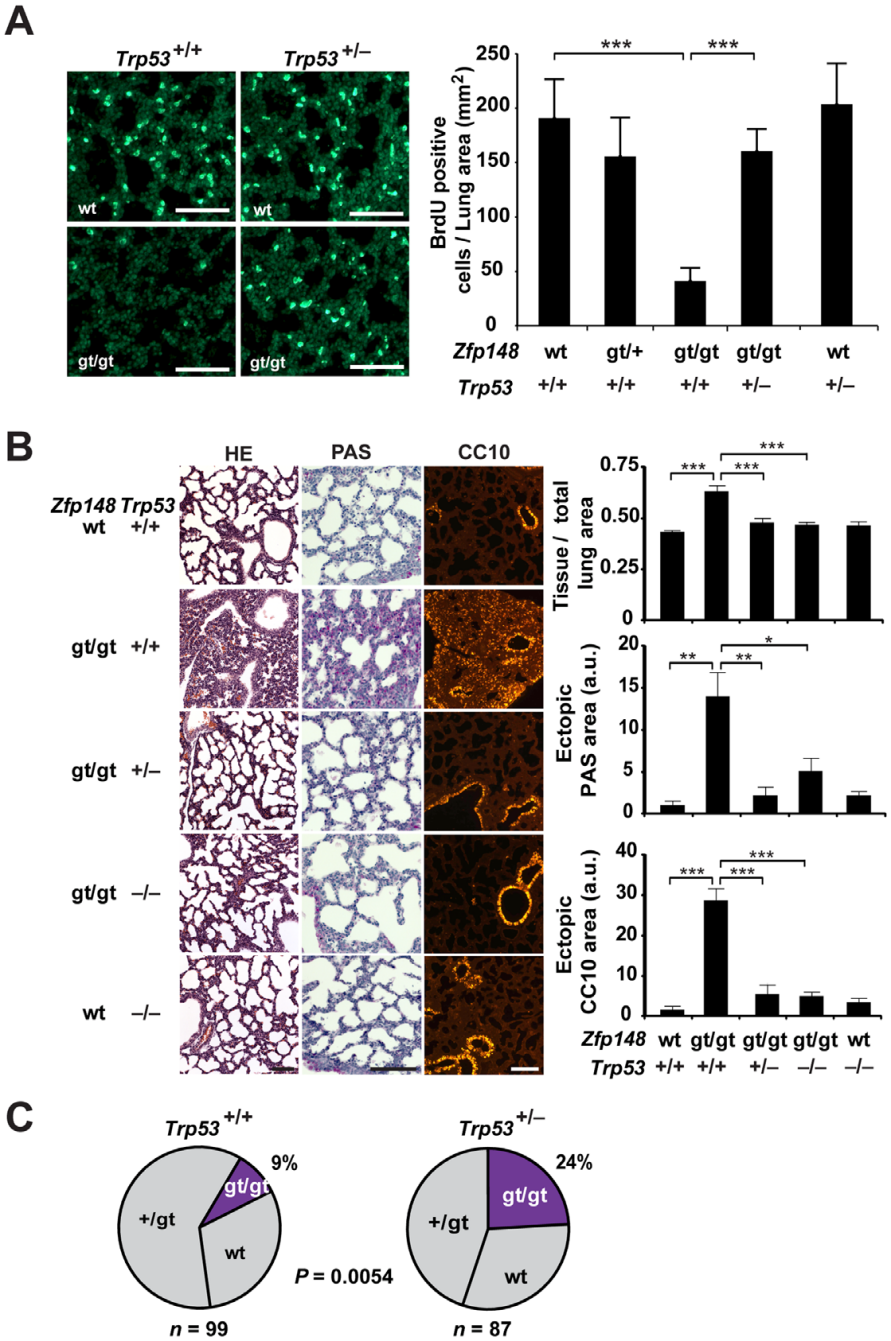
Deletion of one or two copies of *Trp53* rescued *Zfp148^gt/gt^* mice from proliferation arrest, respiratory distress and neonatal lethality. (A) Photomicrographs showing BrdU labeling of E19.5 lung from wt, *Zfp148^gt/gt^*, *Zfp148^gt/gt^Trp53^+/−^* and *Trp53^+/−^* mice. Graphs show quantification of BrdU positive cells per lung area (*n* = 6 wt, 7 *Zfp148^+/gt^*, 7 *Zfp148^gt/gt^*, 5 *Zfp148^gt/gt^Trp53^+/−^*, 4 *Trp53^+/−^*). (B) Photomicrographs showing lung morphology (hematoxylin and eosin; HE) and glycogen content (periodic acid-Shiff; PAS) and CC10 immunofluorescence in P1 lungs from *Zfp148^+/+^Trp53^+/+^* (*n* = 10), *Zfp148^gt/gt^Trp53^+/+^* (*n* = 9), *Zfp148^gt/gt^Trp53^+/−^* (*n* = 9), *Zfp148^gt/gt^Trp53^−/−^* (*n* = 5), and *Zfp148^+/+^Trp53^−/−^* (*n* = 10) mice, respectively. Graphs show quantification of mean tissue area per total lung area, PAS-positive area with bronchioles excluded, and CC10-positive area with bronchioles excluded. (C) Distribution of *Zfp148* genotypes of P1 pups of intercrosses between *Zfp148^+/gt^Trp53^+/+^* and *Zfp148^+/gt^Trp53^+/−^* mice. Scale bars, 100 µm. *P<0.05, **P<0.01, ***P<0.001.

We further investigated if the proliferation arrest and maturation defect of *Zfp148^gt/gt^* lungs are mediated by p53. Indeed, loss of one copy of *Trp53* restored cell proliferation in *Zfp148^gt/gt^* lungs, as judged by BrdU incorporation (Fig. 7A). Moreover, loss of one or two copies of *Trp53* prevented the thickening of the primary septae, glycogen accumulation, and ectopic CC10 expression in *Zfp148^gt/gt^* lungs (Fig. 7B). The loss of one copy of *Trp53* also rescued *Zfp148^gt/gt^* mice from neonatal lethality (Fig. 7C).

### Antioxidant Rescue of Respiratory Distress and Neonatal Lethality of *Zfp148^gt/gt^* Mice

**Figure 8:**
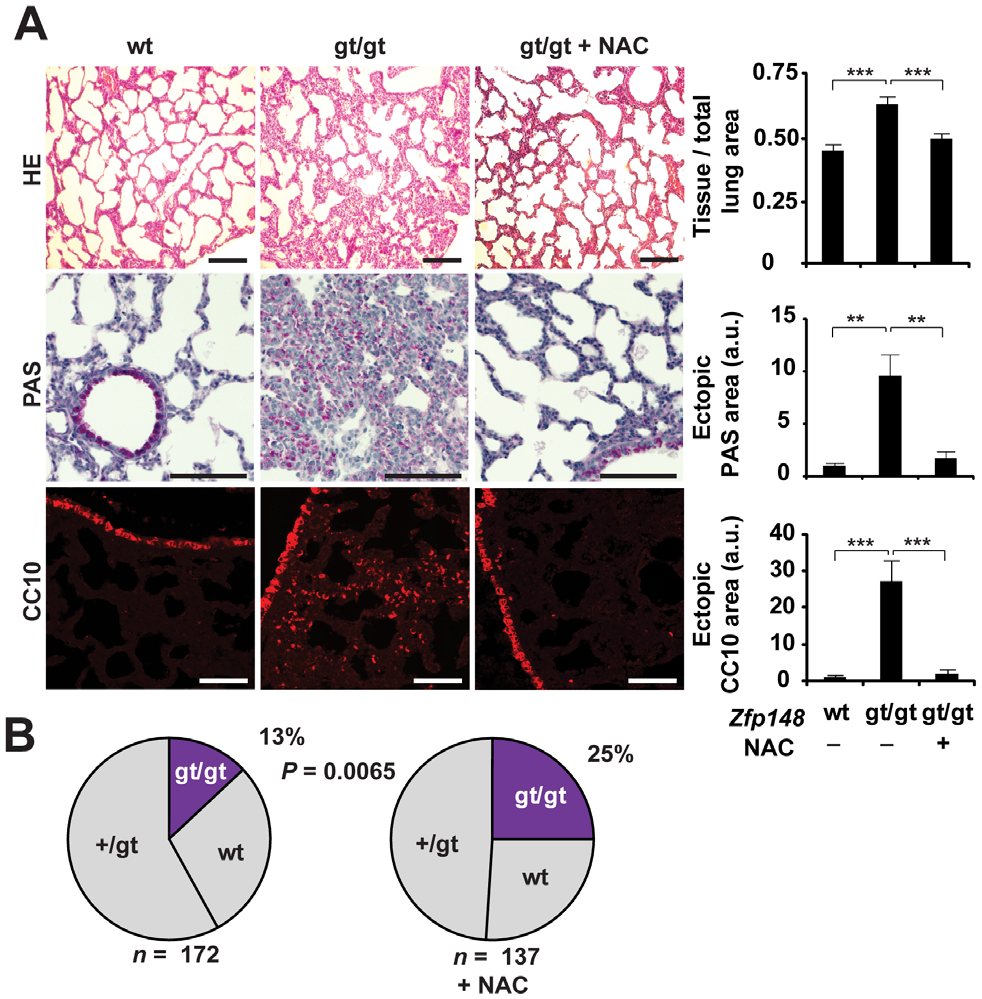
Antioxidant rescue of defect lung maturation and neonatal lethality in *Zfp148^gt/gt^* mice. (**A**) Photomicrographs showing lung morphology (hematoxylin and eosin; HE) and glycogen content (periodic acid-Shiff; PAS) and CC10 immunofluorescence in P1 lungs from *Zfp148^+/+^*, *Zfp148^gt/gt^*, and NAC treated *Zfp148^gt/gt^* mice, respectively (*n* = 6). Graphs show quantification of mean tissue area per total lung area, PAS-positive area with bronchioles excluded, and CC10-positive area with bronchioles excluded. (**B**) Distribution of *Zfp148* genotypes of P1 pups of intercrosses between *Zfp148^+/gt^* mice with and without NAC treatment. Scale bars, 100 µm. **P<0.01, ***P<0.001.

To determine if the respiratory distress and neonatal lethality of *Zfp148*-deficient mice are triggered by oxidative stress, we added NAC in the drinking water of pregnant females of heterozygous intercrosses. Strikingly, NAC treatment of pregnant females normalized lung morphology and prevented glycogen accumulation and ectopic CC10 expression of *Zfp148^gt/gt^* offspring, and rescued them from neonatal death (Fig. 8A, B).

## Discussion

In this study, we show that *Zfp148* deficiency in mice causes respiratory distress and neonatal lethality, and that both of those effects were rescued on *Trp53^+/−^* and *Trp53^−/−^* genetic backgrounds and by antioxidant treatment. Mechanistic analyses showed that *Zfp148* deficiency lowered the threshold for p53 activation under oxidative conditions thus disrupting cell proliferation and structural maturation of the prenatal lung.

*Zfp148* deficiency prevented structural maturation of the prenatal lung without affecting type II cell differentiation or surfactant production. In this respect, *Zfp148*-deficient mice differ from other transgenic models with similar structural maturation defects, since those models also display impaired epithelial cell differentiation or lack of surfactant [25]–[33]. Thus, *Zfp148* deficiency may unravel novel aspects of lung development.

Our data demonstrate that *Zfp148* deficiency activates p53, and that structural maturation defects in *Zfp148*-deficient lungs are secondary to unchecked p53 activity. The mechanism by which p53 disrupts lung maturation is, however, not clear. Proliferation arrest of pulmonary cells probably contributes to the phenotype, but effects of p53-activation on cytoskeletal remodeling or cell migration, as described in [34]–[37], may play a role. Effects on cell differentiation are also possible, as judged by the ectopic expression of CC10 and reduced expression of Clara cell biomarker genes. Nevertheless, the rescue of *Zfp148^gt/gt^* lungs upon deletion of *Trp53* or antioxidant treatment shows that *Zfp148* deficiency affects cell stress or stress responses, whereas Zfp148 is dispensable for lung development *per se*.

The results show for the first time that Zfp148 is required for cell proliferation *in vivo*, confirming previous *in vitro* data [8]. Cell proliferation was selectively arrested in prenatal lungs whereas the embryo as a whole developed to term without gross malformations, suggesting that Zfp148 is required for proliferation in certain contexts. The antioxidant rescue of *Zfp148*-deficient lungs and the rescue on *Trp53^+/−^* and *Trp53^−/−^* genetic backgrounds, suggest that the main function of Zfp148 is to protect cells from p53-activation under oxidative conditions. In consistency with those results, *Zfp148* deficiency lowered the threshold for p53-activation by oxygen in MEFs. Although *Zfp148* deficiency may alter redox homeostasis, the previously demonstrated binding between Zfp148 and p53 opens up for the possibility that Zfp148 primarily regulates p53 [14]. Our demonstration of genetic interaction between *Zfp148* and *Trp53* in mice supports this possibility. Formal evidence of the proposed regulation will, however, require generation of Zfp148 mutants with deficient p53-binding. Future experiments should also establish whether concentrations of reactive oxygen species are elevated in *Zfp148*-deficient cells.

Prenatal lungs are particularly sensitive to the loss of Zfp148 and tissue hypoxia could be a contributing factor. Hypoxia increases production of reactive nitrogen or oxygen species in the mitochondria thus generating oxidative stress [38]–[41]. Mice targeted for *Hypoxia-inducible factor 2α* (*Hif2α*), or mice lacking *Hif1a* in airway epithelium, exhibit structural maturation defects reminding of those seen in *Zfp148*-deficient mice with additional effects on epithelial cell differentiation and surfactant production [25], [42]. Hypoxia-inducible factors are only active under hypoxic conditions [43], [44], suggesting that hypoxia is a driving force of prenatal lung maturation. Thus, levels of reactive nitrogen or oxygen species could be elevated in pulmonary cells at this stage, compared to other cells in the embryo, making them vulnerable to *Zfp148* deficiency.

The defects of *Zfp148*-deficient lungs are reminiscent of clinical findings in newborns with bronchopulmonary dysplasia (BPD). BPD is a developmental disorder with disrupted postnatal growth of the distal lung caused in part by oxidative damage inflicted by oxygen supplementation of preterm infants [45], [46]. Our finding, that Zfp148 protects the prenatal lung from p53-induced proliferation arrest in response to oxidative stress in mice, opens up for the possibility that Zfp148 plays a role in the oxidative damage of preterm lungs in BPD. Studies in primates and mice show increased expression of p53 and p21 after postnatal oxygen supplementation [47]–[49], supporting that possibility.

The physiological role of Zfp148 has remained uncertain due to inconsistent results of gene targeting experiments in mice. A previous attempt to target *Zfp148* resulted in sterile chimeric mice and it was suggested that haploinsufficiency for *Zfp148* blocks germ cell differentiation [13]. Our study clearly demonstrates that *Zfp148*-heterozygous mice are fertile and that *Zfp148* is dispensable for early mouse development. One possible explanation for this discrepancy is that the targeting strategy used by Takeuchi and co-workers (deletion of exon 9) generates a dominant negative phenotype. Alternatively, the *129P2/OlaHsd* embryonic stem cell line that we used is more robust and maintains pluripotency better upon *Zfp148* deficiency, compared to the *AB1* strain used in Takeuchi’s study. We conclude that in the current study, *Zfp148* mRNA levels in *Zfp148^gt/gt^* cells were reduced by >95% and the Zfp148 protein was undetectable.

Another study showed that mice generated from the same *Zfp148* gene-trap mutation used in our study die at E8.5–E10.5 with unclosed neural tubes and anaemia [12]. We observed this phenotype in embryos of the first generation (F1) intercrosses. Importantly, this phenotype co-segregated with a gene-trap vector but not with the recombined *Zfp148* locus (Supporting Information Fig. S3A–C). Moreover, the gene-trap vector was propagated to 79% of the brown offspring (F1 mice) of crosses between chimeric mice and C57Bl/6 mice. This deviates from the expected Mendelian distribution (62 of 78 mice, *P* = 1.5 ×10^−7^, binomial distribution) but is consistent with a second integration of the gene-trap vector in the embryonic stem cell line. Thus, the embryonic death is not caused by *Zfp148* deficiency.

In summary, we show that *Zfp148* is required for structural maturation of the prenatal lung by preventing oxidative stress–dependent p53 activity during the saccular stage of lung development. The result demonstrates for the first time that Zfp148 plays a critical role for cell cycle progression *in vivo*, and establishes Zfp148 as a novel factor in mammalian lung development.

## Supporting Information

Figure S1 **Respiratory distress in** ***Zfp148*** **-deficient mice is not caused by apoptosis.** TUNEL and cleaved caspase 3 (cl-CASP3) staining in lungs of P1 *Zfp148* ^gt/gt^ and wt mice (*n* = 6). Sections of thymus from a 3-week-old wild-type mouse were used as a positive control.
(TIF)

Figure S2 **Wt and** ***Zfp148*** **-deficient MEF proliferation on** ***Trp53*** **^+/−^ or** ***Trp53*** **^−/−^ backgrounds (** ***n*** **= 3).**
(TIF)

Figure S3 **Integration of a second gene-trap in the XB878 ES cell clone.** (**A**) Dissection of 21 E9.5 embryos of F1 generation intercrosses identified a proportion of embryos with unclosed neural tubes and variable degrees of additional defects similar to those described in [12]. Importantly, these mice had at least one intact *Zfp148* allele as judged by PCR-amplification of a DNA fragment spanning the gene-trap insertion site of the *Zfp148* locus (*Zfp148*wt PCR). Moreover, the gene-trap vector was propagated to 79% of the brown offspring (F1 mice) of crosses between chimeric mice and C57Bl/6 mice, which deviates from the expected Mendelian distribution (62 of 78 mice, *P* = 1.5 x 10^−7^, binomial distribution) but is consistent with the presence of two gene-trap alleles in the injected ES-cells. (**B, C**) Dorsal view of E9.5 embryos exhibiting unclosed neural tubes (arrows).
(TIF)

Table S1 **HPLC and mass spec analysis of surfactant lipids from P1 lungs from wt and** ***Zfp148*** **^gt/gt^ mice, first two columns are means for respective genotypes (** ***n*** **= 4) followed by** ***p*** **-value.**
(PDF)

Table S2 **Primer List.**
(PDF)
