# Supplementary figures and images for: Zfp148 Deficiency Causes Lung Maturation Defects and Lethality in Newborn Mice That Are Rescued by Deletion of p53 or Antioxidant Treatment
Source: PLoS One. 2013 Feb 6;8(2):e55720. doi: 10.1371/journal.pone.0055720 (PMC3566028; doi:10.1371/journal.pone.0055720)

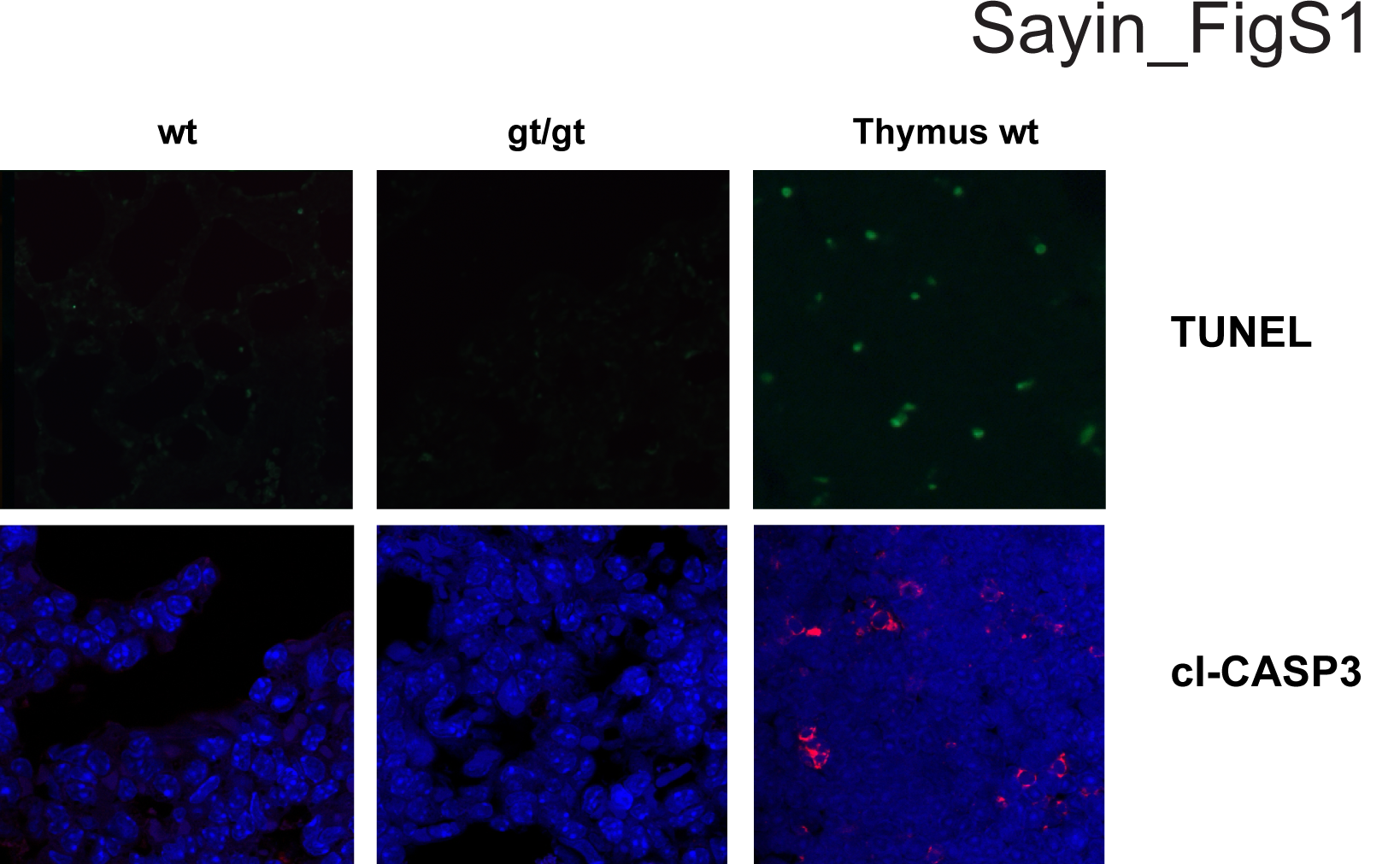

Supplement: Figure S1 — Respiratory distress in Zfp148 -deficient mice is not caused by apoptosis. TUNEL and cleaved caspase 3 (cl-CASP3) staining in lungs of P1 Zfp148 gt/gt and wt mice (n = 6). Sections of thymus from a 3-week-old wild-type mouse were used as a positive control. (TIF) [file pone.0055720.s001.tif]

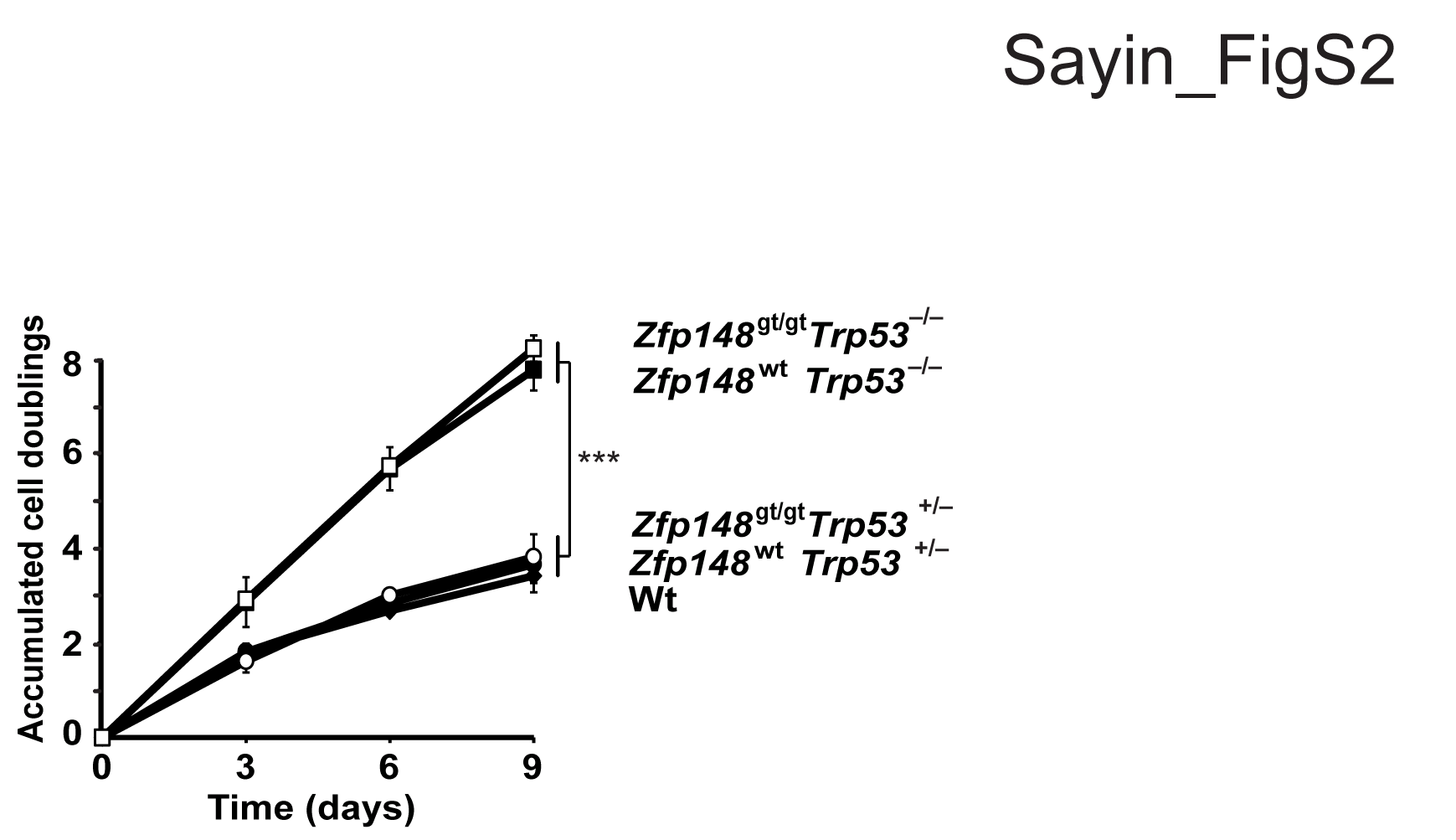

Supplement: Figure S2 — Wt and Zfp148 -deficient MEF proliferation on Trp53 +/− or Trp53 −/− backgrounds ( n = 3). (TIF) [file pone.0055720.s002.tif]

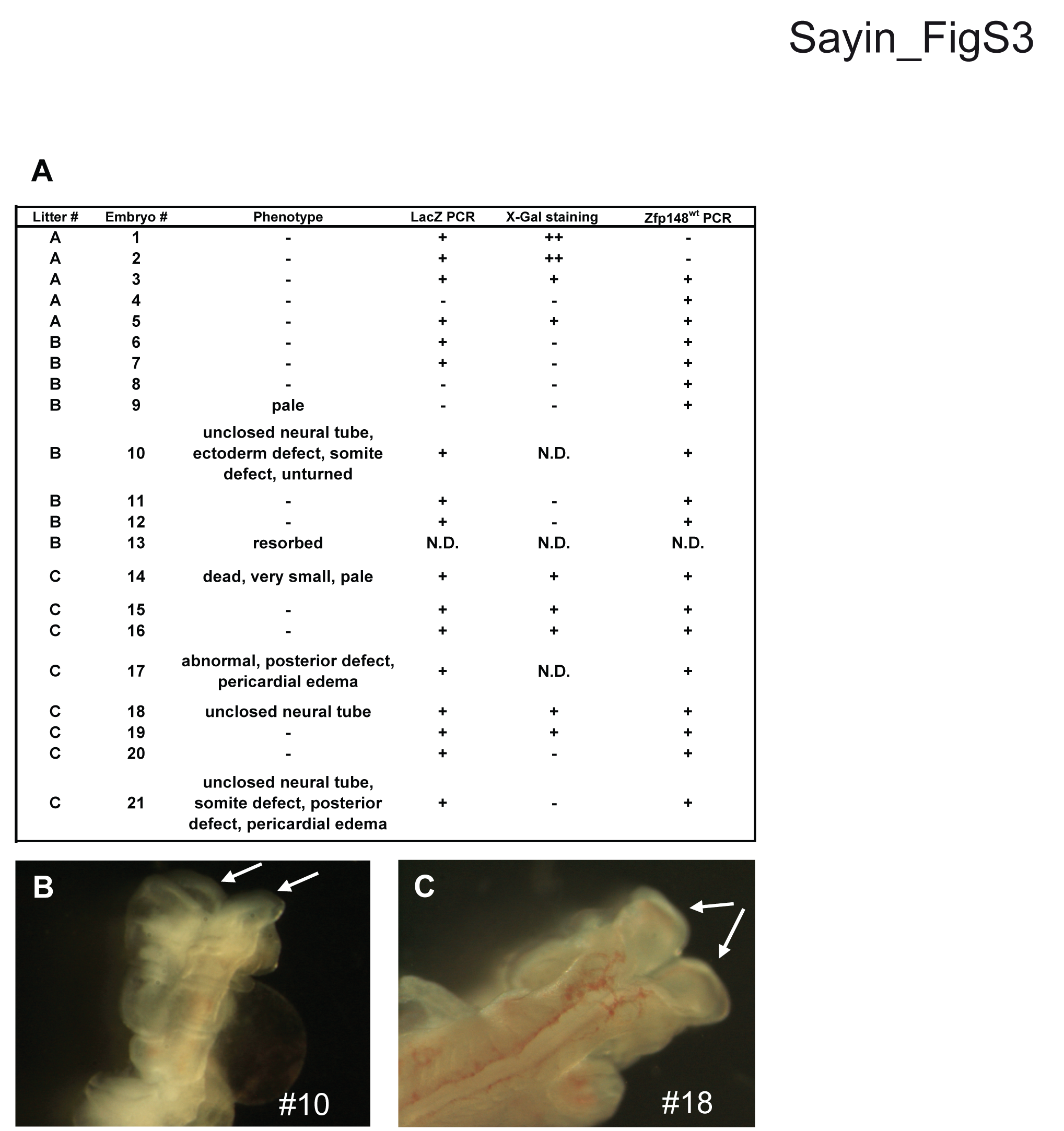

Supplement: Figure S3 — Integration of a second gene-trap in the XB878 ES cell clone. (A) Dissection of 21 E9.5 embryos of F1 generation intercrosses identified a proportion of embryos with unclosed neural tubes and variable degrees of additional defects similar to those described in [12]. Importantly, these mice had at least one intact Zfp148 allele as judged by PCR-amplification of a DNA fragment spanning the gene-trap insertion site of the Zfp148 locus (Zfp148wt PCR). Moreover, the gene-trap vector was propagated to 79% of the brown offspring (F1 mice) of crosses between chimeric mice and C57Bl/6 mice, which deviates from the expected Mendelian distribution (62 of 78 mice, P = 1.5 x 10^−7^, binomial distribution) but is consistent with the presence of two gene-trap alleles in the injected ES-cells. (B, C) Dorsal view of E9.5 embryos exhibiting unclosed neural tubes (arrows). (TIF) [file pone.0055720.s003.tif]
