# Supplementary material for: Zfp148 Deficiency Causes Lung Maturation Defects and Lethality in Newborn Mice That Are Rescued by Deletion of p53 or Antioxidant Treatment
Source: PLoS One. 2013 Feb 6;8(2):e55720. doi: 10.1371/journal.pone.0055720 (PMC3566028; doi:10.1371/journal.pone.0055720)
Supplement: Table S1 — HPLC and mass spec analysis of surfactant lipids from P1 lungs from wt and Zfp148 gt/gt mice, first two columns are means for respective genotypes ( n = 4) followed by p -value. (PDF) [file pone.0055720.s004.pdf]

Supplemental Table S1

Amount (nmol/mg tissue)		*Zfp148*^{wt}	*Zfp148*$^{gt/gt}$	*P*-value
	Triglyceride	3,5	2,8	0,78
	Unesterified cholesterol	9,1	8,8	0,11
	Phosphatidylcholine	15,5	15,2	0,72
	Phosphatidylethanolamine	1,9	1,8	0,17
	Phosphatidylglycerol	1,0	1,1	0,77
Phospholipid composition				
	PC 14:0/14:0	0,2	0,2	0,41
	PC 14:0/16:1	1,0	1,1	0,15
	PC 14:0/16:0	7,3	7,1	0,24
	PC 16:0/16:2	0,5	0,5	0,31
	PC 16:1/16:1	1,5	1,9	0,36
	PC 14:0/18:1	0,6	0,6	0,85
	PC 16:0/16:1	26,0	27,2	0,32
	**PC 16:0/16:0**	**24,4**	**22,8**	**0,89**
	PC 16:1/18:2	0,3	0,4	0,65
	PC 16:0/18:2	4,0	3,9	0,66
	PC 16:1/18:1	1,9	2,0	0,93
	PC 16:0/18:1	17,7	17,2	0,21
	PC 16:0/18:0	1,0	1,0	0,5
	PC 16:0/20:4	3,2	3,3	1,0
	PC 16:0/20:3	0,5	0,4	0,3
	PC 18:1/18:2	1,1	1,1	0,1
	PC 18:0/18:2	0,9	1,0	0,3
	PC 18:1/18:1	2,0	1,9	0,1
	PC 18:0/18:1	2,0	2,1	0,3
	PC 18:0/18:0	0,1	0,1	0,8
	PC 16:0/22:6	0,6	0,7	0,3
	PC 16:0/22:5	0,5	0,4	0,5
	PC 18:1/20:4	0,8	0,7	0,3
	PC 16:0/22:4	0,4	0,4	0,7
	PC 18:0/20:4	1,1	1,2	0,8
	PG 14:0/16:1	0,2	0,2	0,6
	PG 14:0/16:0	2,1	1,9	0,6
	PG 16:1/16:1	0,7	0,8	0,6
	PG 14:0/18:1	0,4	0,5	0,8
	PG 16:1/16:0	14,2	14,1	0,4
	PG 16:0/16:0	12,8	11,5	0,6
	PG 16:1/18:2	0,5	0,6	0,7
	PG 16:0/18:3	0,2	0,2	0,3
	PG 16:1/18:1	3,1	3,9	0,9
	PG 16:0/18:2	5,9	5,8	0,5
	PG 16:1/18:0	0,4	0,5	0,3

PG 16:0/18:1	24,0	24,0	0,9
PG 16:0/18:0	0,4	0,4	0,5
PG 16:0/20:4	6,6	5,6	0,4
PG 18:2/18:2	0,4	0,4	0,8
PG 16:0/20:3	0,8	0,6	0,6
PG 18:1/18:2	3,5	4,1	0,9
PG 16:0/20:2	0,5	0,3	0,1
PG 18:0/18:2	0,6	0,6	0,8
PG 18:1/18:1	7,2	8,6	0,4
PG 18:1/18:0	1,6	1,6	1,0
PG 16:0/22:6	1,5	1,7	0,9
PG 18:2/20:4	0,4	0,4	0,9
PG 16:0/22:5	2,0	1,6	0,4
PG 18:1/20:4	2,8	3,0	0,6
PG 16:0/22:4	1,0	0,9	0,7
PG 18:1/20:3	0,7	0,6	0,3
PG 18:0/20:4	1,0	0,9	0,8
PG 18:1/20:2	0,4	0,4	0,1
PG 18:1/20:1	0,2	0,2	0,2
PG 18:1/22:5	1,8	1,9	0,4
PG 18:0/22:6	0,3	0,5	0,5
PG 18:1/22:4	0,7	0,7	0,7
PG 18:0/22:5	0,2	0,3	0,9
PE 16:1/16:1	0,6	0,7	0,2
PE 16:0/16:1	6,2	6,2	0,0
PE 16:0/16:0	0,9	0,7	0,7
PE 16:1/18:2	1,2	1,1	0,3
PE 16:0/18:2	3,5	3,3	1,0
PE 16:1/18:1	3,8	3,6	1,0
PE 16:0/18:1	15,0	13,6	0,8
PE 16:1/18:0	1,7	1,8	0,6
PE 16:1/20:4	1,2	1,5	0,7
PE 16:0/20:4	9,9	11,0	1,0
PE 16:0/20:3	0,9	0,8	0,7
PE 18:1/18:2	3,3	3,2	0,6
PE 18:0/18:2	1,9	2,2	0,0
PE 18:1/18:1	9,3	8,8	0,5
PE 18:0/18:1	6,5	6,5	0,8
PE 18:0/18:0	1,0	1,7	0,3
PE 18:1/20:4	7,0	7,7	1,0
PE 16:0/22:4	2,9	2,4	0,6
PE 18:0/20:4	14,1	15,1	0,7
PE 18:1/20:3	1,0	0,8	0,2
PE 18:0/20:3	1,5	1,6	0,0
PE 18:1/22:4	1,7	1,6	0,8
