# Supplementary material for: Zfp148 Deficiency Causes Lung Maturation Defects and Lethality in Newborn Mice That Are Rescued by Deletion of p53 or Antioxidant Treatment
Source: PLoS One. 2013 Feb 6;8(2):e55720. doi: 10.1371/journal.pone.0055720 (PMC3566028; doi:10.1371/journal.pone.0055720)
Supplement: Table S2 — Primer List. (PDF) [file pone.0055720.s005.pdf]

Supplemental Table S2

Genotyping

Allele	Forward primer	Reverse primer	Tm	Amplicon (bp)
$Zfp148^{gt}$	GGCCCGTCATAATTTAGGTTG	TGCTGAGGATGAGGGAGCAG	55	450
$Zfp148^{wt}$	GGCCCGTCATAATTTAGGTTG	ACCGGAAGAAAAAGCAGA	55	579
$Trp53^{tm1Tyj}$	ACAGCGTGGTGGTACCTTAT	TCCTCGTGCTTTACGGTATC	55	525
$Trp53^{wt}$	ACAGCGTGGTGGTACCTTAT	ATAGGTCGGCGGTTCAT	55	375

Verification of insertion of gene trap in intron 4 of *Zfp148*

Description	Forward primer	Reverse primer
RT-PCR exon4 - gene trap	ATGGTCCACGAGGAGACAGT	TGCTGAGGATGAGGGAGCAG
RT-PCR exon4 - exon5	ATGGTCCACGAGGAGACAGT	CGTTGTTTCCGCTTCTTCTT
Genomic PCR 8256 bp - gene trap	GGCCCGTCATAATTTAGGTTG	TGCTGAGGATGAGGGAGCAG
Genomic PCR 8256 bp - 8813 bp	GGCCCGTCATAATTTAGGTTG	ACCGGAAGAAAAAGCAGA

RT-PCR assays

Name	Primer sequence	Tm	Amplicon (bp)
zfp148_exon1_fw	AGCTGCCACAGCAAAAGTTC	55	166
zfp148_exon2_rv	CGCCAGATCACTTGGCTAAA	55	166
zfp148_exon2_fw	TGGTTTGGAAATTTAGCCAAG	55	173
zfp148_exon3_rv	CTTCAAGGAAGGGAATGCTG	55	173
zfp148_exon3_fw	TGGCACAAGTGACTGAGAGG	55	240
zfp148_exon4_rv	ATTACAACCATTGCCCTGGA	55	240
zfp148_exon4_fw	ATGGTCCACGAGGAGACAGT	55	202
zfp148_exon5_rv	CGTTGTTTCCGCTTCTTCTT	55	202
zfp148_exon5_fw	AAGAAGAAGCGGAAACAACG	55	152
zfp148_exon6_rv	TGAATGAAGACATGCCTCTG	55	152
zfp148_exon6_fw	GGATGGATCACTTGGTTTGAA	55	179
zfp148_exon7_rv	TCTCGTGTCTCTGAAGCAGGT	55	179
zfp148_exon7_fw	CGAAAAACCGTTTCAATGTAGTC	55	156
zfp148_exon8_rv	CTTTTGTGCCTTTCCATGTG	55	156
zfp148_exon8_fw	CCATTTCGCTGTGATGAATG	55	170
zfp148_exon9_rv	GTCGTGATTTTCATGGCACA	55	170
p16 fw	GTACCCCGATTCAGGTGATG	55	101
p16 rv	GGAGAAGGTAGTGGGGTCCT	55	101

TaqMan assays (Applied Biosystem)

Name	Gene Symbol	Assay ID
Zfp148	Zfp148	Mm00711990_m1
SP-A	Sftpa1	Mm00499170_m1
SP-B	Sftpb	Mm00455681_m1
SP-C	Sftpc	Mm00488144_m1
SP-D	Sftpd	Mm00486060_m1
T1alpha	Pdpn	Mm00494716_m1
Aqp5	Aqp5	Mm00437579_m1
CC-10	Scgb1a1	Mm01230908_m1
Pon1	Pon1	Mm00599936_m1
Pecam1	Pecam1	Mm01242584_m1
Acta2	Acta2	Mm01546133_m1
Tie2	Tie2	Mm00443243_m1
eNOS	Nos3	Mm00435217_m1
p21	Cdkn1a	Mm00432448_m1
